# Clinical Radiological and Molecular Profile of a Patient Affected With Multicentric Osteolysis Nodulosis and Arthropathy

**DOI:** 10.7759/cureus.16615

**Published:** 2021-07-25

**Authors:** Eleftherios Mandragos, Dimitris Myrgiotis, Spyridon Strongylos, Yvonne-Mary Papamerkouriou, John Michelarakis

**Affiliations:** 1 2nd Orthopaedic Department, General Children's Hospital “Panagiotis & Aglaia Kyriakou”, Athens, GRC; 2 2nd Orthopaedic Department, General Children's Hospital “Panagiotis & Aglaia Kyriakou, Athens, GRC

**Keywords:** multicentric osteolysis, mona, arthropathy, joint contractures, mmp2, matrix metalloproteinase 2

## Abstract

Multicentric Osteolysis Nodulosis and Arthropathy (MONA) is an ultra-rare multisystem autosomal recessive disorder characterized by progressive osteolysis, subcutaneous nodules and developing arthropathy. The characteristic radiological signs combined with symptoms resembling juvenile idiopathic arthritis (JIA) set the diagnosis, which is established either by measuring matrix metalloproteinase-2 (MMP-2) enzyme activity through electrophoresis (zymography) or genomic testing. We report the clinical and radiographic findings of a 14-year-old girl with molecularly proven MONA, who presented with painless osteolytic changes of the feet and upper extremities and developed hip arthritis. To this day, no specific therapy has been identified with proven long term relief and control of the disease progression.

## Introduction

Multicentric Osteolysis Nodulosis and Arthropathy (MONA, MIM #259600), also known by the terms nodulosis-arthropathy-osteolysis syndrome (NAO) and Torg-Winchester syndrome, is an infrequently described skeletal dysplasia [1,2]. In 1969 Winchester et al. first described skeletal changes in two sisters born of first-cousin consanguineous parents [3]. However, later genomic analysis of fibroblasts revealed mutations within the matrix metalloproteinase-14 gene, implying clinical overlap between two different “vanishing bone” syndromes. Therefore, the term “Winchester syndrome” is obsolete. MONA is characterized by excessive bone resorption (particularly of the carpal and tarsal bones), generalized osteoporosis, painless subcutaneous fibrocollagenous nodules on the palms and soles and progressive arthropathy manifested predominantly as joint swelling, pain, contractures and stiffness [4]. These phenotypic manifestations are caused by loss of function mutations within the MMP2 gene encoding matrix metalloproteinase-2 (MMP-2) [1].

## Case presentation

**Figure 1:**
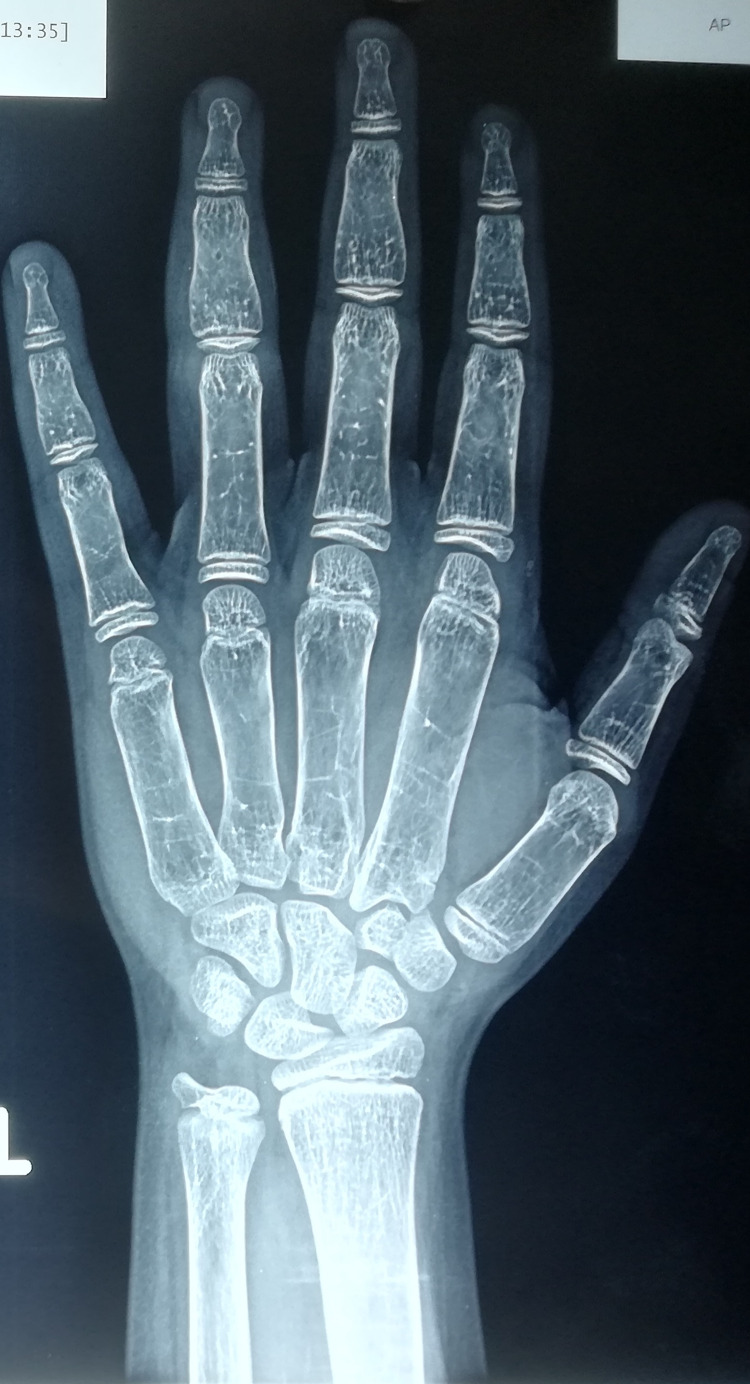
Decreased bone mineralization with cortical thinning at the age of 10.

**Figure 2:**
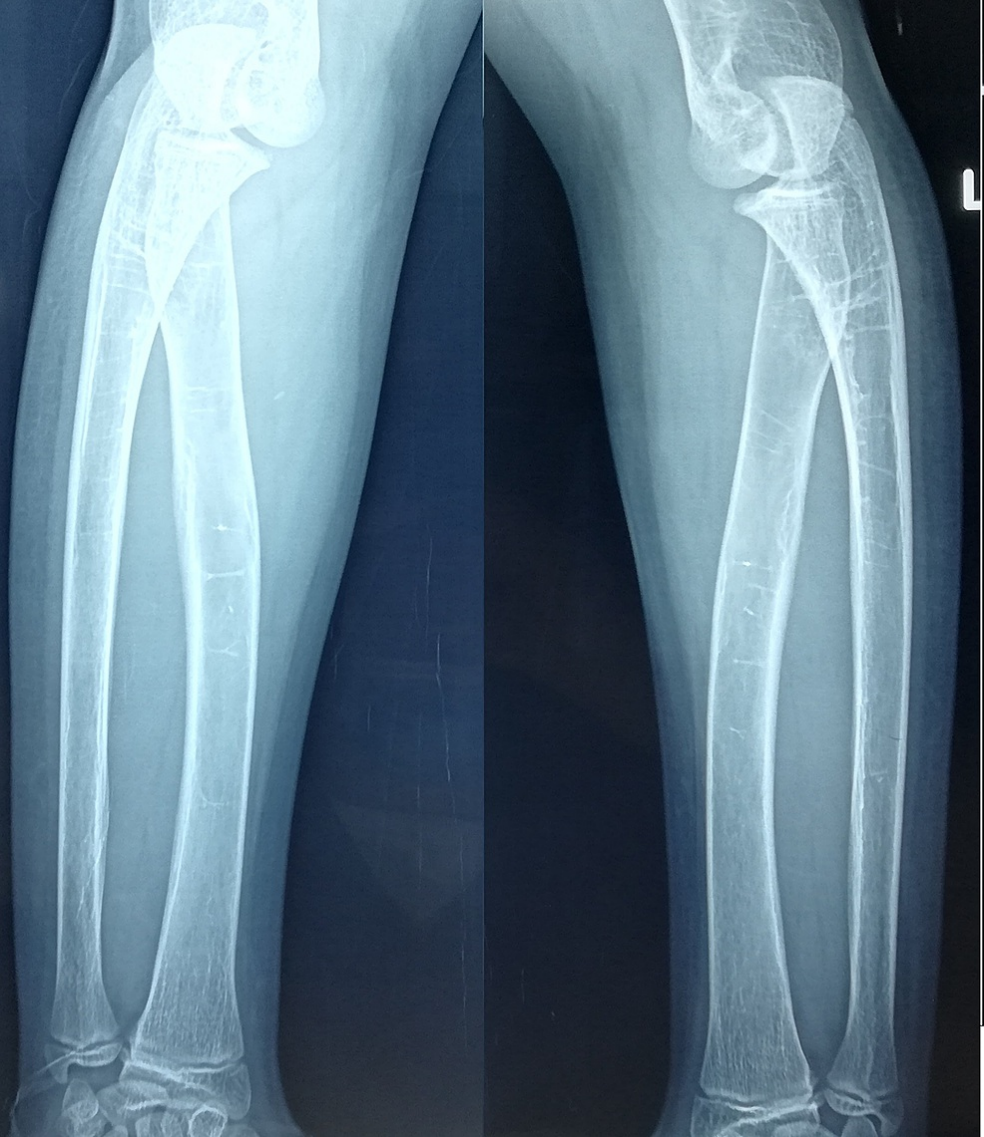
Signs of osteopenia and bone deformity of the distal humerus and proximal ulna at the age of 12.

**Figure 3:**
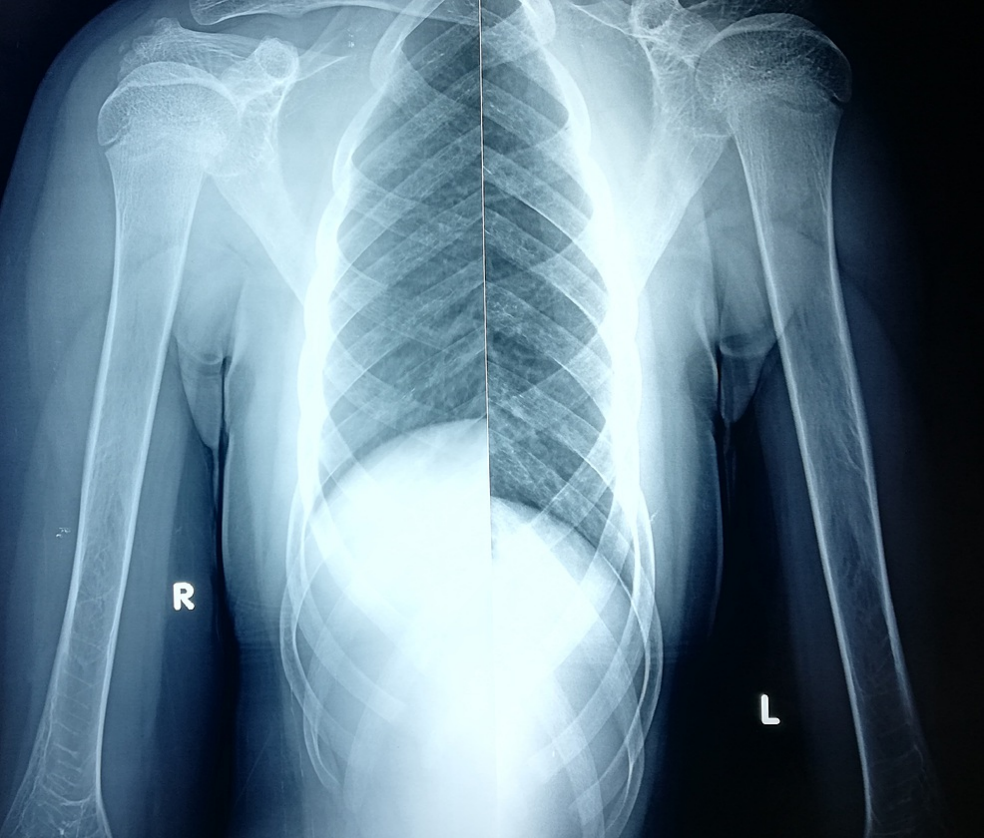
Signs of bone deformity can be appreciated at the right distal humerus at 12 years old.

**Figure 4:**
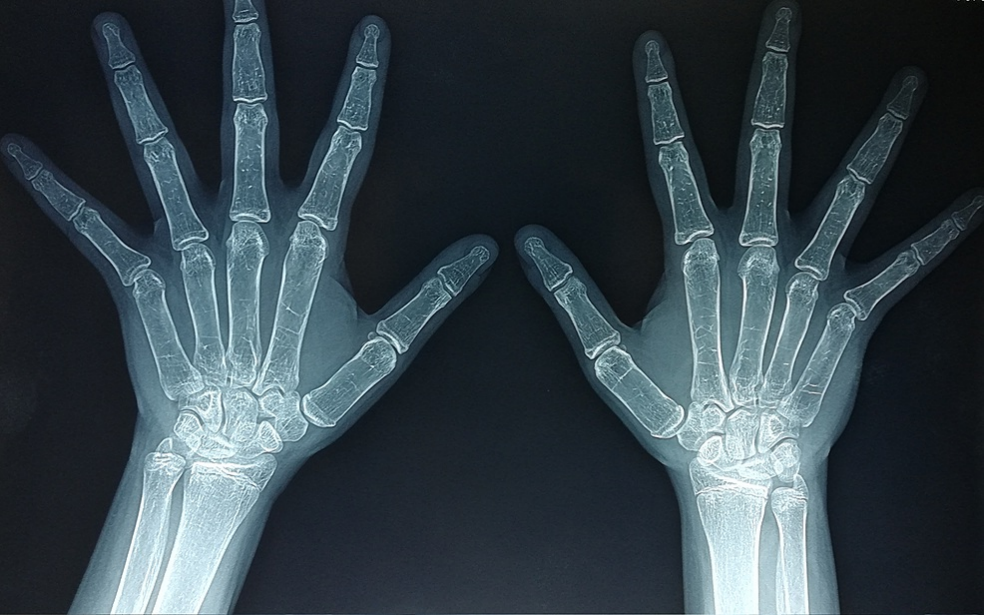
Bilateral osteolysis at the age of 12.

**Figure 5:**
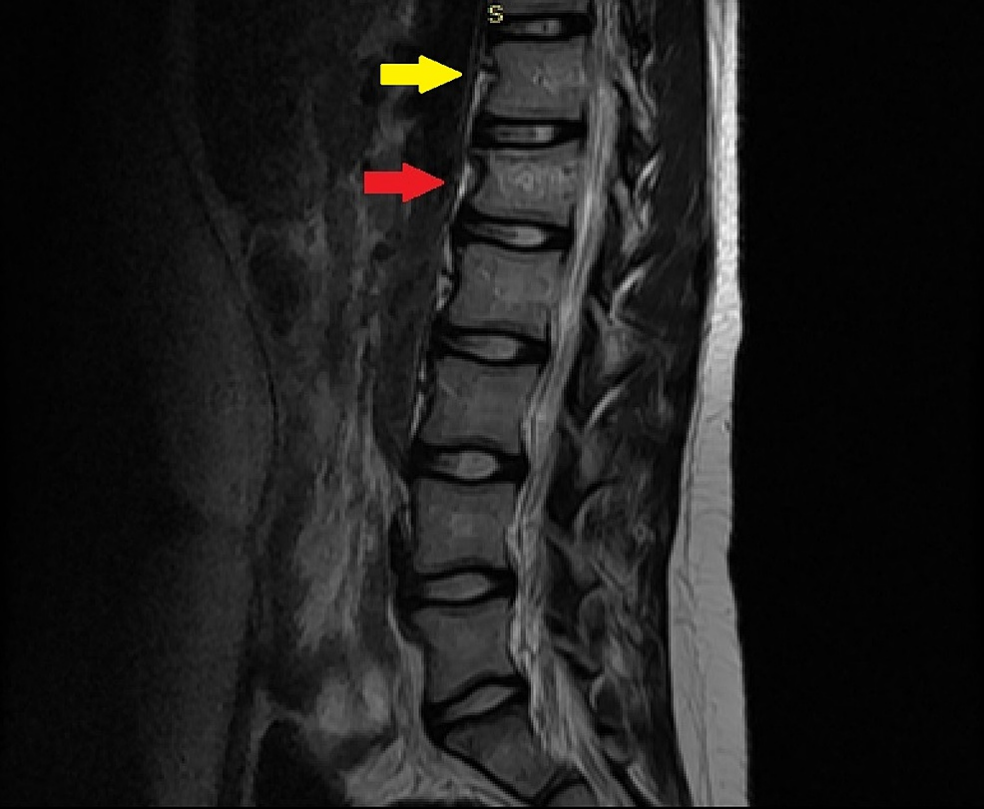
T2 sagittal MR image of the lumbar spine. Wedge fracture of the T12 (yellow arrow) and L1 vertebra with associated diffuse marrow edema (red arrow).

We report the case of a 14-year-old female only child who was born following an uneventful pregnancy. She had a negative family history concerning MONA manifestations. Early childhood was unremarkable apart from a diaphyseal clavicle fracture following an injury treated conservatively and uneventfully. Bilateral hand osteopenia was detected as an incidental finding following a visit at the local hospital for a low energy injury of the right ring finger at the age of 10 (figure 1). The patient was reassured, and regular follow up was advised. Due to ongoing generalized bone pain, a skeletal survey was performed at the age of 12, which revealed the forearm and distal humerus (figure 2, 3) and worsening osteopenia of the hands (figure 4). After a high energy injury, she sustained an uncomplicated wedge fracture of the T12 and L1 vertebrae (MRI scan in figure 5) and was referred to our department for further investigation and treatment.

**Figure 6:**
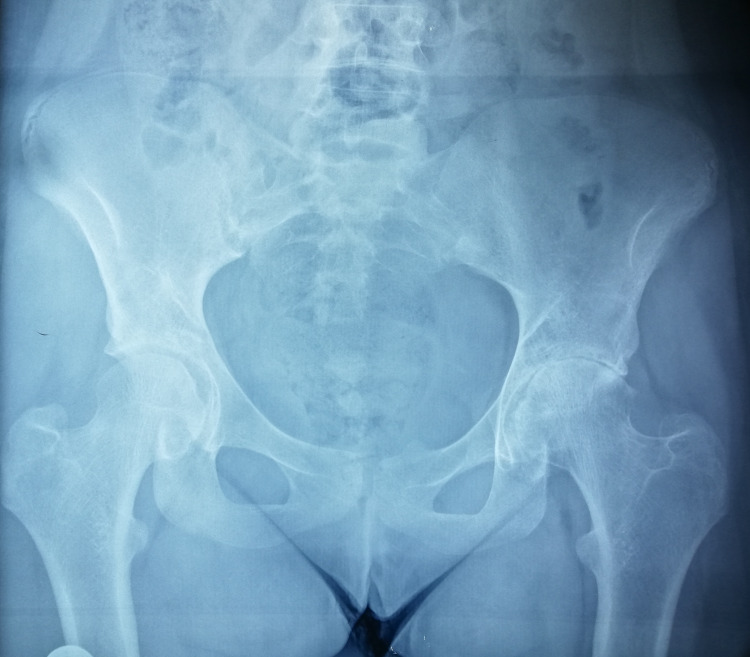
Joint space narrowing of the left hip at the age of 12.

**Figure 7:**
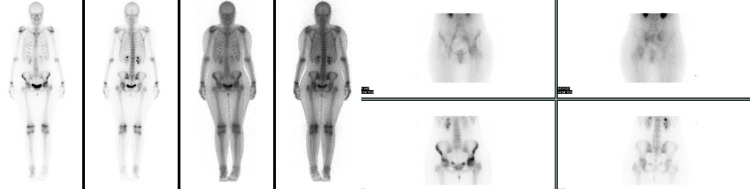
Anterior and posterior planar images from Technetium 99m whole-body bone scan reveal significantly increased uptake in the left hip (mostly anteriorly), increased uptake in the ipsilateral patellofemoral joint and diffusely decreased uptake in the left malleoli-foot.

**Figure 8:**
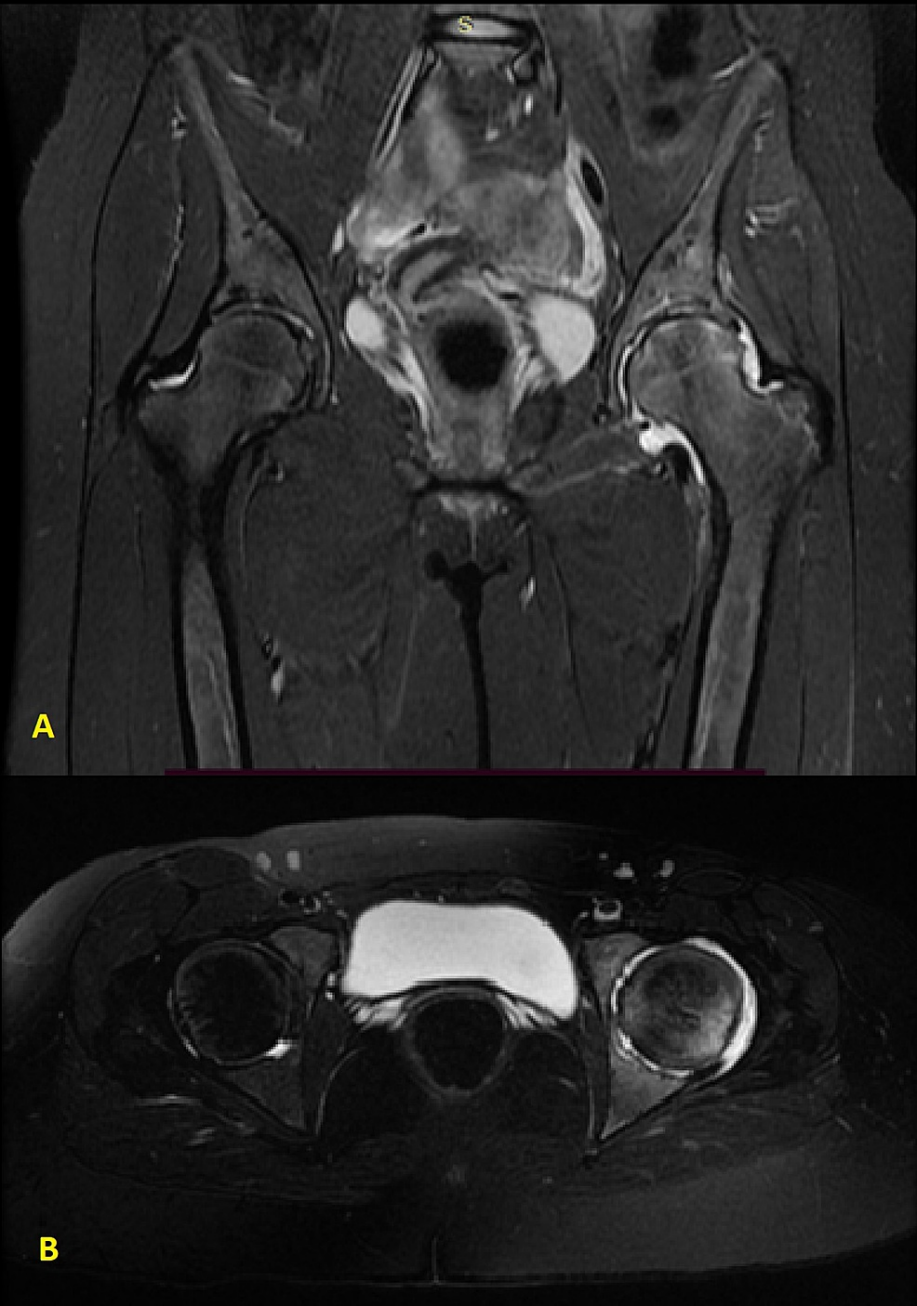
A. Coronal T1-weighted MRI with an abnormal signal of the left epiphysis and synovial thickening. B. Axial T2-weighted MRI showing increased intraarticular fluid and epiphyseal oedema.

The patient had been complaining of left hip pain months before the injury. Although musculoskeletal assessment revealed painful restriction of hip rotation, she completely lacked other characteristic non-osseous involvement. She had no coarsening of facial features. Gum hypertrophy, subcutaneous nodules and skin hyperpigmentation were also absent. Intelligence was normal. Ophthalmic examination and cardiac evaluation, including echocardiogram, were normal. As juvenile idiopathic arthritis (JIA) was considered part of the differential diagnostic approach, antinuclear antibody (ANA) and rheumatoid factor (RF) were ordered and were negative. Erythrocyte sedimentation rate (ESR), c-reactive protein (CRP) and complete blood count (CBC) were unremarkable throughout. An anterior-posterior radiograph of the pelvis demonstrated joint space narrowing and bone irregularity of the left hip (figure 6). Bone scintigraphy with (99m)Tc-MDP demonstrated significantly increased uptake in the left acetabulum, representing osteomyelitis or arthritis. Abnormal uptake was also noted in the asymptomatic ipsilateral knee (slightly increased) and foot (diffusely decreased) (figure 7). MRI arthrography of the left hip detected epiphyseal edema, an abnormal signal of the epiphysis, synovial thickening and an abnormal amount of joint fluid (figure 8). Whole-body bone densitometry (DEXA) was within the lower normal limits (z-score =-1.4). However, the lumbar spine assessment was positive for osteopenia (z-score =-2.4), and therefore calcium - Vitamin D3 supplements were recommended. The 24-hour urine collection revealed hypercalciuria. Genomic analysis of the patient with the whole exome sequencing technique showed compound heterogenicity for MMP2. The patient carries the exon 3 point mutation c.529G>A, which causes the amino acid substitution p.(Glu177Lys) and has a negative effect on RNA splicing. Another mutation was detected in exon 9 (c.1462_1464del), resulting in the amino acid deletion p.(Phe488del). DNA sequencing confirmed that each parent had one of the pathogenic variants. Hip joint aspiration was negative for infection, and a subsequent intraarticular steroid injection had excellent results with symptom alleviation for months.

## Discussion

Patients with MONA disease are often children of consanguineous parents, but there is no such relation in this case [5]. The small joints are typically affected first, and patients invariably present with pain and reduced range of motion of their hands and feet [6]. Shortening of digits and camptodactyly occurs with age as well as progressive osteolysis [7]. Knees, hips, wrists, ankles and elbows may be deformed, and the disease is associated with bowing of the long bones and talipes equinovarus (table 1). Chronic joint destruction results in paradoxical increased passive range of motion and excessive skin folds in the hands and feet [8]. Although height is normal in little children, short stature may develop later due to combined osseous and joint deformities [9]. One-third of the patients suffer from cardiac defects such as mitral valve prolapse, ventricular septal defect and outflow tract abnormalities [10]. Kyphoscoliosis and vertebral anomalies are observed in 50% of the patients. Reduced bone mineral density is a constant feature that increases the risk for fractures. Some individuals have prominent eyes, large cornea, and corneal opacities at presentation, but no vision impairment has been reported. Other non-osseous features of this condition are facial dysmorphism, bulbous nose, flat nasal bridge hypertelorism, hirsutism, gingival hypertrophy, skin hyperpigmentation. Cognitive abilities are not affected [11].

**Table 1:** Selected clinical features of MONA

Clinical feature	%
Osteolysis	100%
Osteopenia	100%
Arthropathy	100%
Subcutaneous nodules	80%
Contractures	100%
Ophthalmic anomalies	43%
Cardiac anomalies	22%
Coarse face	75%
Dental abnormalities	50%

The rarity of the syndrome limits the available data on its natural history. The majority of the affected children appear healthy at birth, and the first signs of the disease appear between six months and six years (median is three years). However, the range of onset can vary considerably from birth to 11 years. Children initially present with pain and swelling of hands and feet. Most individuals develop the face during the disease and distinct subcutaneous firm nodules and other non-skeletal manifestations. Early acceleration of bone destruction may lead to debilitating arthropathy in adolescence when the progression of the disease slows down [12].

Radiographically, generalized osteopenia begins at the hands and the feet with cortical thinning of well-formed metacarpals, metatarsals, and phalanges [6]. As the disease progresses, the tubular bones lose their normal contour and become widened. Osteolysis is evident in advanced cases with narrowing and erosions of the interphalangeal joints, the collapse of the carpal rows, shortening and destruction of the tarsus [12]. Arthropathy may eventually extend to the hips, knees, elbows, spine and sacroiliac joints with similar changes of milder severity [4]. Long bones with thin cortices and associated osteopenia have also been reported. Osteopenia or osteoporosis of the entire skeletal system is more pronounced in older patients [13]. A bone scan may help locate multiple sites of involvement and guide further MRI imaging [12].

The diagnosis is established either with molecular genetic testing or with gelatin-zymographic analysis of MMP-2 in blood samples of selected children with radiographic and clinical findings consistent with MONA [14]. Genomic sequencing in patients reveals biallelic pathogenic mutations in the responsible gene. Zymography of the MMP-2 compared to controls will show a complete loss of enzyme activity. There are no widely accepted diagnostic criteria [15].

Early in the disease, patients are often erroneously diagnosed with systemic or polyarticular JIA and are subsequently subjected to ineffective treatment with anti‐inflammatory drugs, systemic corticosteroids and immunomodulating drugs. A high level of suspicion is crucial for the correct diagnosis, appropriate management and early genetic counselling for the family and the at-risk relatives. JIA68uukkmfr,6r7 can be excluded based on progressive loss of motion, worsening joint pain, absence of cardiac malformations, and a lack of positive family history [16]. On the contrary, the pain subsides or disappears at later stages of MONA. Radiographs that show widened tubular bones and the typical changes of multicentric osteolysis in a patient with fibrocollagenous nodules and coarse facial features should suggest MONA [17]. Other disorders that share phenotypic findings with MONA and should be considered in the differential diagnosis are mucopolysaccharidoses, hyaline fibromatosis syndrome and familial expansile osteolysis [1].

MONA is inherited in an autosomal recessive manner and is caused by inactivating mutations in the matrix metalloproteinase 2 (MMP2) gene (MIM no.120360), which is located on chromosome 16q21 and contains 13 exons [3]. To date, 23 inactivating mutations from 28 families have been reported in the literature. Patients carried biallelic or homozygous private mutations throughout the gene sequence [1]. Pathogenic variants of concern were deletions, missense, nonsense, and splice-site mutations [1].

**Figure 9:**
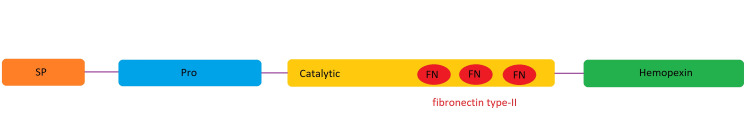
MM2 structure. Signal Peptide (SP), Pro domain, Catalytic domain, fibronectin type-II(FN), hemopexin like domain.

The pathogenetic background of MONA is the MMP2 gene deficiency leading to complete loss of the MMP-2 activity, also known by the term gelatinase A or 72kDa type IV collagenase, the most widely expressed metalloproteinase [4]. It consists of four domains (Figure 9), with the catalytic domain being significant for its highly conserved region, both across species and different types of matrix metalloproteinases [18]. Upon activation, MMP-2 regulates osteoblastic and osteoclastic activity promotes pro-inflammatory cytokines and affects cell adhesion, proliferation, differentiation, and apoptosis. The thin balance between bone formation and resorption is disrupted with MMP-2 defects that cause turnover dysfunction through collagen denaturation, angiogenesis and TGF-1 signalling [8]. Supporting evidence is provided from MMP2‐null mice who demonstrate joint destruction and decreased bone mineral density [16].

 

 

As a sporadic disease with only 43 cases confirmed and with various combinations of biallelic genes, a relation between specific mutations and phenotypic findings or prognosis has not yet been established [17,18].

To this day, there is no specific alleviating treatment. Steroids, immunosuppressive therapy and bisphosphonates do not benefit from disease progression or symptom improvement and should be withheld due to their side effects. Pain medication is occasionally proven unsuccessful [19]. Management is limited to supportive care, including physical therapy and aims in delaying the appearance of contractures. Surgical release of contractures and corrective osteotomies are controversial [1,8].

## Conclusions

Early recognition of MONA is important to protect the patient from potentially harmful treatment and avoid referrals for unnecessary investigations. Patients are often diagnosed with JIA as there are overlapping symptoms and radiographic signs. However, the progression of osteopenia, the sequence of the arthropathy location and the characteristic clinical features should raise a strong suspicion for this disease. Further investigation on the complex interaction of MMP-2 with the connective tissue turnover is warranted to enlighten the pathophysiology of the disease and guide treatment.
